# A bioactive ginsenoside alleviates hepatocellular ferroptosis induced by oxidized phospholipid accumulation in nonalcoholic steatohepatitis

**DOI:** 10.1002/imt2.70114

**Published:** 2026-03-03

**Authors:** Yibo Zong, Guo Long, Tiantian Gu, Pan Huang, Yong Tian, Wenwu Xu, Xiheng Hu, Dazun Shi, Lizhi Lu, Tao Zeng

**Affiliations:** ^1^ State Key Laboratory for Quality and Safety of Agro‐Products, Zhejiang Key Laboratory of Livestock and Poultry Biotech Breeding, Institute of Animal Husbandry and Veterinary Science Zhejiang Academy of Agricultural Science Hangzhou China; ^2^ National Clinical Research Center for Geriatric Disorders, Department of Liver Surgery, Xiangya Hospital Central South University Changsha China; ^3^ School of Medicine Jiangsu University Zhenjiang China; ^4^ National Engineering Research Center of Personalized Diagnostic and Therapeutic Technology, Department of Urology, Xiangya Hospital Central South University Changsha China; ^5^ Department of Gynaecology, Xiangya Hospital Central South University Changsha China

## Abstract

Schematic representation showing that 20 (S)‐Rg3 improves mouse NASH. 20(S)‐Rg3 reduces OxPLs levels and ameliorates ferroptosis in the liver of NASH mice. Mechanistically, 20(S)‐Rg3 activates SPOP. Meanwhile, SPOP attenuates the ATF3‐mediated transcriptional repression of NUPR1 by enhancing the K48‐linked ubiquitination of ATF3. Upregulated NUPR1 rescues redox balance and reduces lipid peroxidation, ultimately inhibiting hepatocellular ferroptosis.

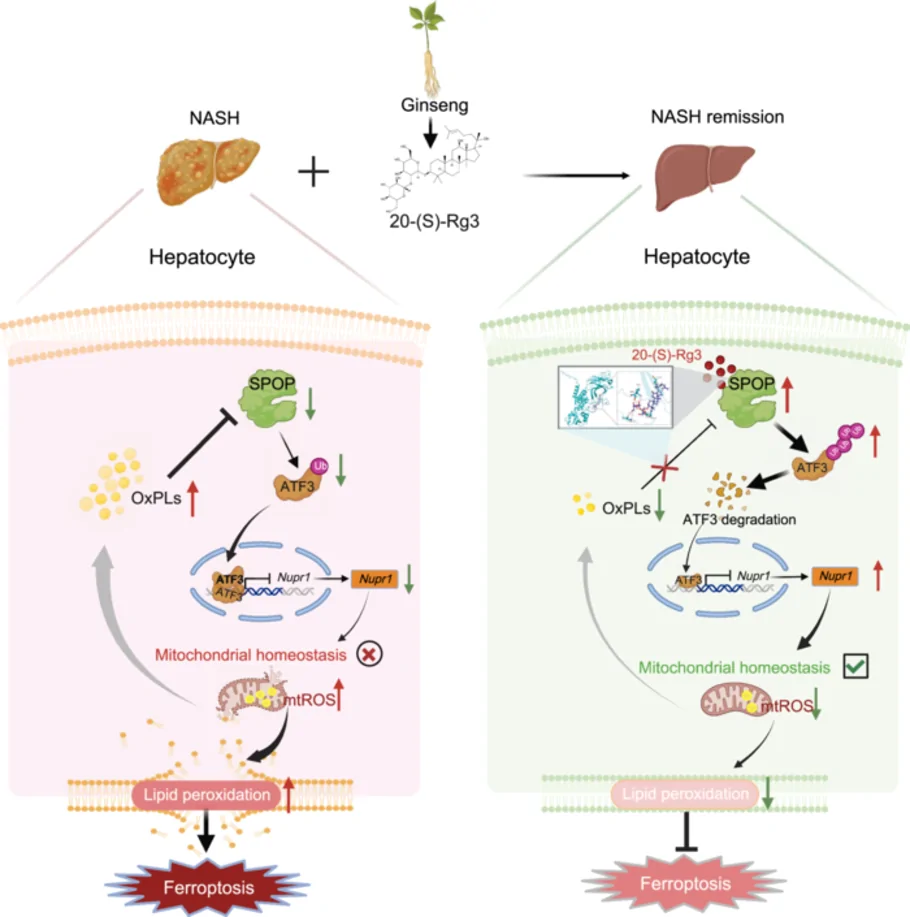

Nonalcoholic steatohepatitis (NASH) affects approximately 25%–30% of the global population and represents the most prevalent chronic liver disease worldwide [1]. Despite its growing clinical burden, effective pharmacological therapies for NASH remain limited, underscoring an urgent need to elucidate its underlying pathogenic mechanisms and to identify actionable therapeutic targets [2, 3]. Ginsenoside Rg3 has been widely reported to exert hepatoprotective effects in preclinical models; however, its specific role and mechanistic contribution to the initiation and progression of NASH have not been fully defined [4, 5].

Oxidized phospholipids (OxPLs) have emerged as bioactive lipid mediators with potent pro‐inflammatory and pro‐atherogenic properties in metabolic diseases [6]. By modulating intracellular signaling pathways and transcriptional programs, OxPLs can induce cellular stress responses and cell death [7, 8]. Accumulating evidence suggests that excessive oxidative stress promotes ferroptosis through OxPLs accumulation, linking lipid peroxidation to regulated cell death [9]. Notably, OxPLs are markedly elevated in liver tissue and circulation in multiple NASH mouse models as well as in NASH patients [10]. However, because OxPLs are primarily derived from non‐enzymatic lipid peroxidation, effective strategies for their neutralization are currently lacking, and their precise causal role in NASH pathogenesis remains largely undefined [6].

Here, we demonstrate that 20 (S)‐ginsenoside Rg3 (Rg3) effectively attenuates hepatic OxPLs accumulation and suppresses ferroptosis in mouse models of NASH. Mechanistically, Rg3 activates the E3 ubiquitin ligase speckle‐type POZ protein (*Spop*), promoting ubiquitination and degradation of activating transcription factor 3 (ATF3). This relieves ATF3‐mediated transcriptional repression of nuclear protein 1 (*Nupr1*), a key regulator of mitochondrial homeostasis, thereby limiting mitochondrial dysfunction, lipid peroxidation, and ferroptotic cell death in hepatocytes. Together, this study reveals a causal link between OxPLs accumulation and ferroptosis in the pathogenesis of NASH and identifies Rg3 as a promising therapeutic candidate targeting this axis. Moreover, our data suggest that circulating OxPLs levels may serve as a non‐invasive biomarker for stratification and diagnosis of NASH.

## HIGH‐DOSE RG3 IMPROVES HIGH FAT DIET (HFD)‐INDUCED NASH IN MICE

To evaluate the therapeutic potential of Rg3, we established a NASH murine model by feeding mice with a HFD for 24 weeks (Figure 1A). High‐dose Rg3 significantly reduced body weight, liver mass, and the liver‐to‐body weight ratio, while alleviating dyslipidemia as evidenced by decreased hepatic levels of triglycerides (TG), total cholesterol (TC), non‐esterified fatty acids (NEFA), and low‐density lipoprotein cholesterol (LDL‐C) (Figure 1B,C, Figure S1A–E). High‐dose Rg3 also decreased plasma alanine aminotransferase (ALT), aspartate aminotransferase (AST), and lactate dehydrogenase (LDH) (Figure 1D). Histopathological analyses confirmed these protective effects, revealing marked reductions in hepatocyte ballooning, steatosis, and fibrosis (Figure 1E, Figure S1F–I).

**Figure 1:**
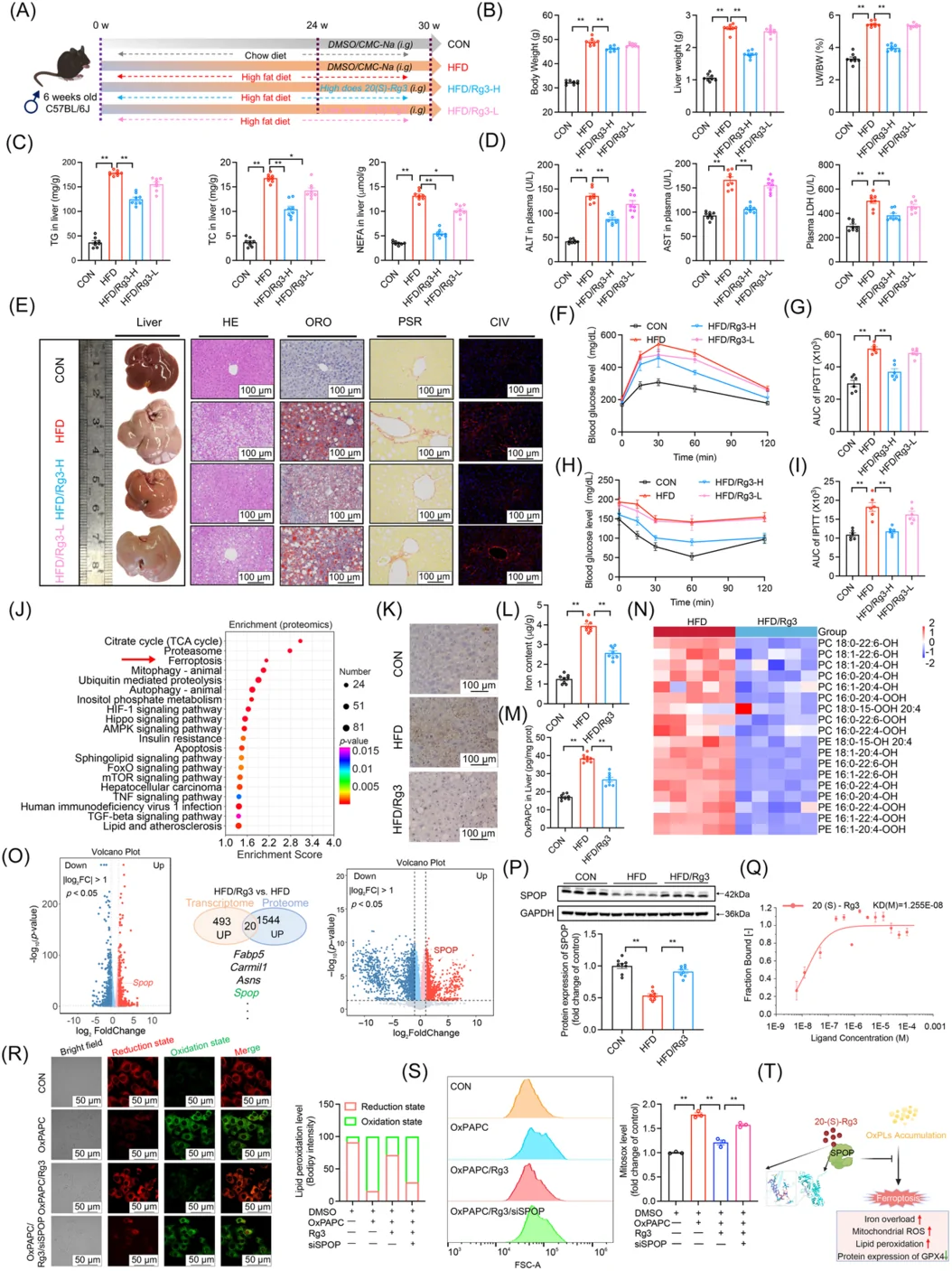
High‐dose Rg3 ameliorates nonalcoholic steatohepatitis (NASH) and upregulates hepatic SPOP expression in high‐fat diet (HFD)‐fed mice. (A) Schematic depicting Rg3 treatment strategy on HFD‐induced NASH mouse model. (B) Body weight, liver weight, and the ratio of liver weight to body weight (LW/BW) in control and treated mice; *n* = 8. (C) Hepatic levels of triglycerides (TG), total cholesterol (TC), and non‐esterified fatty acids (NEFA); *n* = 8. (D) Plasma levels of ALT, AST, and LDH; *n* = 8. (E) Representative hematoxylin and eosin (H&E), oil red O (ORO), picrosirius red (PSR), and collagen IV (CIV) staining of liver sections. (F, G) Intraperitoneal Glucose tolerance test (IPGTT) and area under the curve (AUC) in mice; *n* = 6. (H, I) Intraperitoneal insulin tolerance test (IPITT) and AUC in mice; *n* = 6. (J) KEGG pathway enrichment analysis of mouse hepatic proteomics data; *n* = 4. (K) Representative Perls' Prussian blue staining showing hepatic iron deposition; *n* = 6. (L) Quantification of hepatic iron content of mice; *n* = 8. (M) Hepatic OxPAPC level; *n* = 8. (N) Heatmap showing relative abundance changes of OxPLs species in liver tissues measured by HPLC‐MS oxylipidomics; *n* = 5. (O) Volcano plots of differentially expressed genes (left, HFD/Rg3 vs. HFD) and proteins (right, HFD/Rg3 vs. HFD). (P) Representative Western blot and quantification of SPOP protein expression in liver tissue. (Q) MST test. (R) Representative confocal microscopy images and quantification of lipid peroxidation in AML12 cells stained with BODIPY 581/591; *n* = 3. (S) Flow cytometry analysis of mitochondrial reactive oxygen species (mitoROS) in AML12 cells using MitoSOX staining; *n* = 3. (T) Schematic model illustrating the mechanism by which Rg3 upregulates SPOP expression to suppress lipid peroxidation‐induced ferroptosis in hepatocytes. Data are shown as the mean ± SEM; *p < 0.05, **p < 0.01.

High‐dose Rg3 exerted anti‐inflammatory effects by suppressing key pro‐inflammatory cytokines (TNF‐α, IL‐6, IL‐1β) in both liver tissue and plasma (Figure S1J–O). Given that NASH is closely linked to systemic metabolic dysregulation, we further assessed whole‐body glucose homeostasis [11]. High‐dose Rg3 significantly improved glucose tolerance and insulin sensitivity (Figure 1F–I). Based on these comprehensive beneficial effects, we selected the high‐dose regimen (hereafter referred to as Rg3) for all subsequent mechanistic investigations.

## RG3 ALLEVIATES FERROPTOSIS AND UPREGULATES HEPATIC SPOP EXPRESSION

Multi‐omics profiling consistently identified ferroptosis as a predominantly enriched pathogenic pathway in HFD‐induced NASH, which was markedly suppressed by Rg3 treatment (Figure 1J, Figure S2A–C). Rg3 restored hepatic redox homeostasis disrupted by HFD feeding, as evidenced by increased activities of catalase (CAT) and superoxide dismutase (SOD), elevated glutathione (GSH) levels and GSH/oxidized glutathione (GSSG) ratios, and reduced malondialdehyde (MDA) and GSSG levels (Figure S2D–I).

Ferroptosis, an iron‐dependent form of regulated cell death driven by phospholipid peroxidation, is characterized by iron accumulation and lipid peroxide formation [12]. Rg3 significantly attenuated hepatic iron deposition in HFD‐fed mice (Figure 1K,L, Figure S2J). Notably, total OxPAPC (a representative mixture of oxidized phospholipids (OxPLs)) levels were significantly elevated in the livers of fibrotic mice and cirrhotic patients compared to healthy controls (Figure 1M, Figure S2K). Targeted HPLC–MS oxylipidomics further revealed a broad reduction in multiple OxPLs species following Rg3 treatment (Figure 1N, Figure S2L). In parallel, Rg3 significantly reduced hepatic levels of 4‐hydroxynonenal (4‐HNE), a canonical marker of lipid peroxidation and ferroptosis, and alleviated HFD‐induced mitochondrial damage (Figure S2M,N).

We integrated our multi‐omics datasets and identified the E3 ubiquitin ligase SPOP as a top candidate (Figure 1O). *Spop* has established roles in suppressing inflammation through *Tlr4/Nf‐κb* pathway inhibition [13], and functions as a tumor suppressor in hepatocellular carcinoma [14, 15], yet its role in ferroptosis remains unexplored. Validation experiments confirmed that Rg3 significantly restored *Spop* expression at both the mRNA and protein levels in HFD‐fed mice (Figure 1P, Figure S2O,P), implicating SPOP as a critical regulator in Rg3‐mediated inhibition of ferroptosis.

## SPOP MEDIATES THE PROTECTIVE EFFECTS OF RG3 AGAINST FERROPTOSIS IN AML12 CELLS

We employed an in vitro model using AML12 hepatocytes treated with OxPAPC at concentrations established in previous studies [10]. OxPAPC exposure suppressed SPOP expression in AML12 cells, an effect that was efficiently reversed by Rg3 co‐treatment (Figure S3A,B). OxPAPC induced cell death that was rescued by the ferroptosis inhibitor ferrostatin‐1 (Fer‐1) (Figure S3C), confirming ferroptosis as the primary death mechanism.

Interestingly, micropyrokinetic technique (MST) revealed high‐affinity binding between Rg3 and SPOP (Kd = 12.5 nM), with molecular docking simulations identifying key interaction residues (Figure 1Q, Figure S3D–F). These results suggest there may be a direct binding between SPOP and Rg3.

Functionally, silencing *Spop* largely abolished the cytoprotective effects of Rg3 against OxPLs‐induced cell death (Figure S3G). BODIPY 581/591 C11 staining showed that *Spop* knockdown significantly impaired Rg3‐mediated suppression of lipid peroxidation (Figure 1R). Consistently, Rg3 restored intracellular GSH levels, the GSH/GSSG ratio, and SOD and CAT activities while reducing MDA accumulation following OxPAPC exposure; all of these antioxidant effects were impaired upon *Spop* silencing (Figure S4A–E). Moreover, *Spop* knockdown prevented Rg3‐induced upregulation of the anti‐ferroptotic enzyme GPX4 (Figure S4F) and abolished Rg3‐mediated recovery of NADPH levels in OxPAPC‐treated hepatocytes (Figure S4G). Furthermore, *Spop* depletion abrogated the protective effects of Rg3 on multiple mitochondrial parameters (Figure 1S, Figure S4H–J).

Collectively, these findings establish SPOP as both a potential direct molecular target of Rg3 and an indispensable mediator of its anti‐ferroptotic activity in hepatocytes (Figure 1T).

## SPOP PROMOTES ATF3 UBIQUITINATION AND DEGRADATION TO SUPPRESS FERROPTOSIS

To identify downstream effectors of SPOP, we performed immunoprecipitation followed by mass spectrometry (IP‑MS) to capture SPOP‑interacting proteins in AML12 cells. KEGG revealed significant enrichment of ferroptosis‐related pathways. Integrating these IP‐MS results with our in vivo liver proteomics data identified ATF3 as a prominent SPOP‐interacting protein (Figure 2A,B). While ATF3 has been implicated in hepatic steatosis and glucose dysregulation, its precise role in metabolic liver disease remains controversial [16, 17]. Our results showed that Rg3 treatment reduced OxPAPC‑induced ATF3 protein accumulation (Figure 2C), and ATF3 knockdown partially restored GPX4 expression following OxPAPC exposure (Figure S5A).

**Figure 2:**
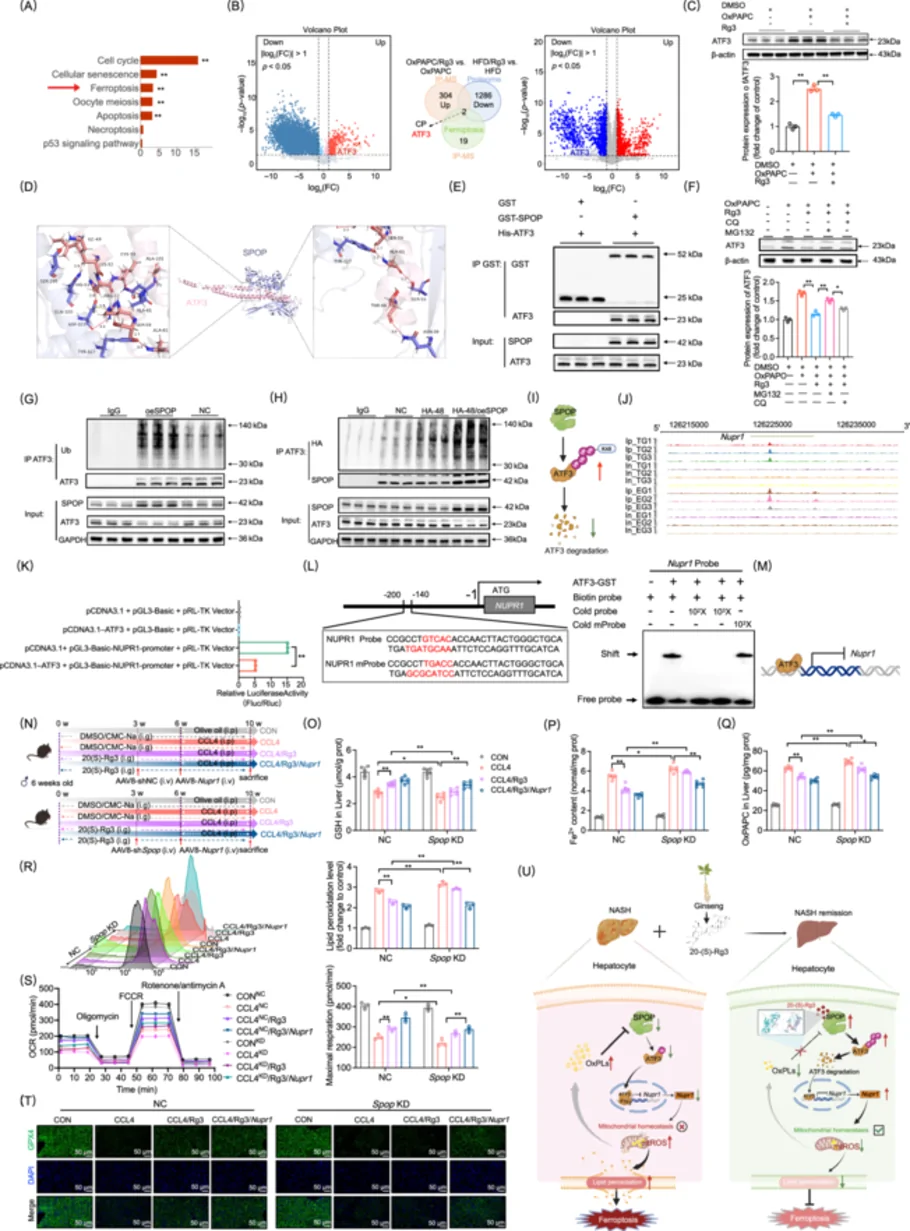
Rg3 Alleviates hepatocyte ferroptosis via the *Spop*/*Atf3*/*Nupr1* axis. (A) KEGG pathway enrichment analysis of immunoprecipitation‐mass spectrometry. (B) Venn diagram (Middle) showing overlap between differentially expressed proteins identified by IP‐MS (Left) and liver proteomics (Right), with ATF3 highlighted as a key SPOP‐interacting candidate. (C) Representative Western blot and quantification of ATF3 protein expression in AML12 cells treated with vehicle, OxPAPC, or OxPAPC plus Rg3. (D) Molecular docking simulation showing the predicted SPOP‐ATF3 protein complex structure and key binding residues. (E) GST pull‐down assay demonstrating direct physical interaction between GST‐tagged SPOP and His‐tagged ATF3 proteins. (F) Western blot analysis of ATF3 protein stability in AML12 cells treated with OxPAPC (24 h) in the presence of vehicle, chloroquine (CQ, 10 μM, autophagy inhibitor), or MG132 (10 μM, proteasome inhibitor). (G) Co‐immunoprecipitation (Co‐IP) of ATF3 followed by Western blot detection of ubiquitin, demonstrating ATF3 ubiquitination in AML12 cells. (H) Detection of K48‐linked polyubiquitination of ATF3 in cells overexpressing *Spop* and transfected with HA‐tagged ubiquitin constructs, assessed by immunoprecipitation with anti‐ATF3 antibody followed by Western blotting with anti‐HA linkage‐specific antibodies. (I) Schematic model illustrating SPOP‐mediated K48‐linked ubiquitination and proteasomal degradation of ATF3 in hepatocytes. (J) Representative ChIP‐seq tracks showing ATF3 binding at the *Nupr1* gene locus in AML12 cells treated with OxPAPC alone (EG) or OxPAPC plus Rg3 (TG), with input DNA as control. (K) Dual‐luciferase reporter assay measuring *Nupr1* promoter activity in 293T cells co‐transfected with *Atf3* expression plasmid or empty vector control. Relative luciferase activity is normalized to Renilla internal control; *n* = 3. (L) Electrophoretic mobility shift assay (EMSA) demonstrating direct binding of ATF3 protein to the *Nupr1* promoter region. (M) Schematic model showing ATF3‐mediated transcriptional repression of *Nupr1* in hepatocytes. (N) Experimental design schematic for Rg3 treatment in CCL4‐induced liver fibrosis model mice with liver‐specific *Spop* knockdown (AAV8‐*shSpop*) and *NUPR1* overexpression (AAV8‐*Nupr1*). (O) Hepatic Reduced glutathione (GSH) levels in CCL4 model mice *n* = 6. (P) Hepatic iron content in CCL4 model mice; *n* = 6. (Q) Hepatic OxPAPC levels in CCL4 model mice; *n* = 6. (R) Flow cytometry analysis of lipid peroxidation in primary hepatocytes from CCL4 model mice stained with BODIPY 581/591 C11; *n* = 3. (S) Mitochondrial bioenergetics profiles of primary hepatocytes from CCL4 model mice measured by Seahorse XF analyzer, showing oxygen consumption rate (OCR) under basal conditions and following sequential injection of oligomycin, FCCP, and rotenone/antimycin A (left) and quantification of maximal respiration (right); *n* = 3. (T) Representative immunofluorescence images and quantification of glutathione peroxidase 4 (GPX4) expression in liver sections from CCL4 model mice; *n* = 6. (U) Summary schematic model illustrating the molecular mechanisms underlying Rg3‐mediated protection against NASH through the *Spop‐Atf3‐Nupr1*‐ferroptosis axis. Data are shown as the mean ± SEM; *p < 0.05, **p < 0.01.

We next confirmed a direct interaction between SPOP and ATF3 using GST pull‐down assays, Co‐IP, and molecular docking analyses, with Rg3 treatment significantly elevating the amount of SPOP bound to its substrate ATF3 in cells (Figure 2D,E, Figure S5B,C). Importantly, SPOP promoted proteasome‐dependent degradation of ATF3 via K48‐linked, but not K63‑linked, ubiquitination (Figure 2F–I, Figure S6A,B). Functionally, overexpression of SPOP effectively counteracted ATF3‑driven ferroptotic responses in AML12 cells (Figure S6C–F).

## *NUPR1* IS A KEY ATF3‑REGULATED MEDIATOR OF HEPATOCYTE FERROPTOSIS AND FIBROSIS

To delineate the transcriptional program governed by ATF3, we performed ATF3 chromatin immunoprecipitation followed by sequencing (ChIP‑seq) in AML12 cells treated with OxPAPC (EG) or OxPAPC in combination with Rg3 (TG) (Figures S7, S8A). ATF3‐bound genes showed significant overrepresentation of ferroptosis‐ and oxidative stress‐related pathways (Figure S8B,C). By intersecting ChIP‑seq data with pathway annotations, we selected four ATF3‑bound genes implicated in oxidative‑stress and ferroptosis, and *Atf3* knockdown rescued gene and protein expression of *Nupr1* and *Slc7a11* (Figures 2J, Figures S8D, 9).

Given previous reports implicating ATF3 in the regulation of *Slc7a11* [18], and the established role of *Nupr1* as a critical suppressor of ferroptosis and regulator of redox homeostasis [19, 20], we focused on *Nupr1* as a candidate downstream effector. Dual‐luciferase reporter assays and electrophoretic mobility shift assays (EMSA) demonstrated that ATF3 acts as a transcriptional repressor of *Nupr1* (Figure 2K–M). Notably, analysis of liver tissues from patients with cirrhosis and iron overload showed elevated ATF3 expression and concomitant downregulation of SPOP and NUPR1 compared with normal controls (Figure S10).

## *NUPR1* RESCUES THE IMPAIRED ANTI‐FERROPTOTIC FUNCTION OF RG3 CAUSED BY *SPOP* KNOCKDOWN IN HEPATOCYTE

To determine whether *Nupr1* mediates Rg3‑dependent suppression of ferroptosis downstream of *Spop*, we examined its function in AML12 and BRL3A hepatocytes. Re‐expression of *Nupr1* rescued the *Spop*‑knockdown‐induced loss of Rg3‐mediated antioxidant effects (Figure S11A–J). Similarly, *Spop* knockdown markedly attenuated the ability of Rg3 to mitigate OxPAPC‑induced mitochondrial damage, an effect that was largely reversed by *Nupr1* overexpression (Figure S11K,L). Seahorse bioenergetics analysis showed that Rg3 restored OxPAPC‑suppressed oxygen consumption rates (OCR). These effects were eliminated by *Spop* knockdown and significantly recovered upon *Nupr1* overexpression (Figure S11M,N). At the protein levels, *Spop* silencing weakened Rg3‐mediated suppression of the pro‐ferroptotic enzyme ACSL4 and induction of the anti‐ferroptotic GPX4, whereas *Nupr1* partially restored these expression changes (Figure S11O,P).

## OVEREXPRESSION OF *NUPR1* RESCUES THE ATTENUATION OF RG3‐MEDIATED ANTI‐FERROPTOTIC EFFECTS CAUSED BY *SPOP* KNOCKDOWN IN MOUSE LIVER

To validate the functional role of *Nupr1* downstream of *Spop* in vivo, we achieved liver‑specific *Spop* knockdown and *Nupr1* overexpression via adenoviral delivery, followed by establishment of liver fibrosis using CCL4 and induction of hepatocyte ferroptosis through tail‑vein injection of OxPAPC (Figure 2N, Figures S12A,B,13A). *Spop* knockdown significantly attenuated hepatoprotective effects of Rg3 (including boosting antioxidant capacity and reducing iron deposition), and robustly restored by *Nupr1* overexpression (Figure 2O,P, Figures S12C–E, 13B–E, 14A–D). Similarly, *Spop* deficiency diminished the capacity of Rg3 to limit hepatic OxPAPC accumulation, whereas expression of *Nupr1* effectively rescued this effect (Figure 2Q, Figures S12F, 13F, 14C). Flow cytometry analysis further confirmed that *Nupr1* overexpression reversed the *Spop*‑knockdown‐induced loss of Rg3‐mediated inhibition of hepatic lipid peroxidation (Figure 2R, Figure S13G).

OCR assays showed that *Nupr1* alleviated the mitochondrial damage induced by *Spop* knockdown (Figure 2S, Figures S12G,H, 13H). At the protein level, *Spop* knockdown reduced Rg3‐mediated suppression of ACSL4 and F4/80, as well as induction of GPX4; these effects were partially recovered by *Nupr1* overexpression (Figure 2T, Figures S12I–K, 13I, 14E,F).

In summary, we identify 20(S)‐ginsenoside Rg3 as a potent therapeutic candidate for NASH, acting through suppression of OxPLs accumulation and inhibition of hepatic ferroptosis (Figure 2U). Our findings define a previously unrecognized *Spop*‐*Atf3*‐*Nupr1* regulatory axis of ferroptosis in NASH progression and provide targeted therapeutic strategies for this disease.

## AUTHOR CONTRIBUTIONS

**Yibo Zong**: Data curation; methodology; writing—original draft. **Guo Long**: formal analysis; writing—original draft; software. **Tiantian Gu**: Methodology; formal analysis. **Pan Huang**: Formal analysis; resources. **Yong Tian**: Methodology; formal analysis. **Wenwu Xu**: Software; investigation. **Xiheng Hu**: Supervision; writing—review and editing; funding acquisition. **Dazun Shi**: Supervision; writing—review and editing; funding acquisition. **Lizhi Lu**: Supervision; writing—review and editing; funding acquisition. **Tao Zeng**: Funding acquisition; writing—review and editing; supervision. All authors have read the final manuscript and approved it for publication.

## CONFLICT OF INTEREST STATEMENT

The authors declare no conflicts of interest.

## ETHICS STATEMENT

All animal experimental procedures were approved by the Institutional Animal Care and Use Committee of Zhejiang Academy of Agricultural Sciences (24ZALAS21). Human liver tissue samples were obtained from Xiangya Hospital, Central South University, China. All procedures involving human specimens were approved by the Ethics Review Committee of Xiangya Hospital (Ethical Approval Number for Human Tissue Samples: 2025040650).

## Supporting information


**Figure S1.** High‐dose Rg3 ameliorates hyperlipidemia and inflammatory injury in NASH mice.
**Figure S2.** Rg3 alleviates ferroptosis and upregulates hepatic SPOP expression.
**Figure S3.** SPOP and Rg3 molecular docking.
**Figure S4.** *Spop* interference impairs the ferroptosis‐resistant effect of Rg3.
**Figure S5.** Co‐IP between SPOP and ATF3.
**Figure S6.** Overexpression of *Spop* ameliorates ATF3‐induced hepatocyte ferroptosis.
**Figure S7.** The distribution of ChIP‐seq peaks and the top five motifs.
**Figure S8.** Enrichment analysis of ATF3 target genes.
**Figure S9.** The effect of *Atf3* interference on the expression of candidate genes.
**Figure S10.** SPOP, NUPR1, and ATF3 expression in the liver of patients with cirrhosis.
**Figure S11.** *Nupr1* rescues the impaired anti‐ferroptotic function of Rg3 caused by *Spop* knockdown in hepatocyte.
**Figure S12.** Morphological detection in mouse CCL4 model.
**Figure S13.** Overexpression of *Nupr1* rescues the attenuation of Rg3‐mediated anti‐ferroptotic effects caused by *Spop* knockdown in mouse OxPAPC model.
**Figure S14.** Morphological detection in mouse OxPAPC model.

**Table S1.** Key reagents in the experiment.

## Data Availability

The raw sequence data have been deposited in the China National Center for Bioinformation /Beijing Institute of Genomics, Chinese Academy of Sciences under the accession numbers: CRA024779 (https://ngdc.cncb.ac.cn/gsa/browse/CRA024779) and CRA024780 (https://ngdc.cncb.ac.cn/gsa/browse/CRA024780). The mass spectrometry proteomics data have been deposited to the ProteomeXchange Consortium (https://www.iprox.cn/page/SSV024.html;url=1769569968620A2FO) via the iProX partner repository with the password cpxN (Project ID: IPX001516700). The data are saved in GitHub (https://github.com/Zyb1224/supplementary-materials.git). Omics data analysis was performed using the OE biological cloud platform (https://cloud.oebiotech.com). Supplementary materials (methods, figures, tables, graphical abstract, slides, videos, Chinese translated version, and update materials) may be found in the online DOI or iMeta Science http://www.imeta.science/.
