# Supplementary material for: A bioactive ginsenoside alleviates hepatocellular ferroptosis induced by oxidized phospholipid accumulation in nonalcoholic steatohepatitis
Source: Imeta. 2026 Mar 3;5(1):e70114. doi: 10.1002/imt2.70114 (PMC13726593; doi:10.1002/imt2.70114)
Supplement: Supplementary file 1 [file IMT2-5-e70114-s002.docx]

**Supporting information to**

# A bioactive ginsenoside alleviates hepatocellular ferroptosis induced by oxidized phospholipid accumulation in nonalcoholic steatohepatitis

# Running title: A bioactive ginsenoside alleviates hepatocellular ferroptosis

Yibo Zong^1^, Guo Long^2^, Tiantian Gu^1^, Pan Huang^3^, Yong Tian^1^, Wenwu Xu^1^, Xiheng Hu^4^, Dazun Shi^5^*, Lizhi Lu^1^*, Tao Zeng^1^*

^1^State Key Laboratory for Quality and Safety of Agro-Products, Zhejiang Key Laboratory of Livestock and Poultry Biotech Breeding, Institute of Animal Husbandry and Veterinary Science, Zhejiang Academy of Agricultural Science, Hangzhou 310021, China

^2^National Clinical Research Center for Geriatric Disorders, Department of Liver Surgery, Xiangya Hospital, Central South University, Changsha 410008, China

^3^School of Medicine, Jiangsu University, Zhenjiang 212013, China

^4^National Engineering Research Center of Personalized Diagnostic and Therapeutic Technology, Department of Urology, Xiangya Hospital, Central South University, Changsha 410008, China

^5^Department of Gynaecology, Xiangya Hospital, Central South University, Changsha 410008, China.

*Correspondence: zengtao4009@126.com (Tao Zeng); lulizhibox@163.com (Lizhi Lu); shidazun@csu.edu.cn (Dazun Shi)

## Supplementary methods

## Animals and experimental design

In the first set of animal experiments (Figure 1A), male C57BL/6J mice (6 weeks old) were housed under specific pathogen-free conditions at a controlled temperature (22–24°C) with a 12-h light/dark cycle. Mice were randomly assigned to four groups: control (CON), fed a standard chow diet and administered vehicle (0.5% carboxymethylcellulose, CMC) by daily oral gavage; HFD group, fed an HFD (60% kcal from fat; Research Diets, D12492) for 24 weeks to induce hepatic steatosis and fibrosis, with daily vehicle gavage; HFD + Low-dose Rg3 group (HFD/Rg3-L), fed an HFD as above and treated with Rg3 (Rg3: 20 mg/kg body weight/day) by oral gavage for an additional 6 weeks; and HFD + High-dose Rg3 group (HFD/Rg3-H), fed an HFD as above and treated with Rg3 (Rg3: 40 mg/kg body weight/day) by oral gavage for an additional 6 weeks.

In the second set of animal experiments (Figure 2N, S13A), mice were treated with Rg3 (40 mg/kg body weight/day) by intragastric gavage throughout the experimental period. Liver-specific SPOP knockdown (SPOP KD) was achieved by a single tail vein injection of AAV serotype 8 encoding shRNA targeting SPOP (AAV8-shSPOP; 1×10^¹¹^ viral genome (vg) per mouse; shRNA sequences listed in Table S1). At week 6 of Rg3 treatment, hepatic overexpression of NUPR1 was induced by single tail vein injection of AAV8-NUPR1 (1×10^¹¹^ vg per mouse). Both AAV8-shSPOP and AAV8-NUPR1 were designed and produced by Gene Pharma (Shanghai, China).

To induce ferroptosis and liver injury, OxPAPC- and CCL4- challenged models were established. For the OxPAPC model, mice received tail vein injections of OxPAPC (4 mg/kg body weight, once every other day) for 3 weeks. For the fibrosis model, mice were intraperitoneally injected with 10% (v/v) CCL4 in olive oil at a dose of 50 μL per 10 g body weight, three times weekly for 4 weeks. At the end of the experiments, mice were euthanized, and liver tissues and blood samples were collected for downstream analyses. All animal experimental procedures were approved by the Institutional Animal Care and Use Committee of Zhejiang Academy of Agricultural Sciences (24ZALAS21).

### Human specimens

Human liver tissue samples were obtained from Xiangya Hospital, Central South University, China. All procedures involving human specimens were approved by the Ethics Review Committee of Xiangya Hospital (Ethical Approval Number for Human Tissue Samples: 2025040650).

### Preparation and administration of 20(S)-ginsenoside Rg3 (Rg3)

Rg3 (purity > 98%, Chengdu Must Bio-Technology) was dissolved in 0.5% CMC before administration. Vehicle or Rg3 was administered daily via oral gavage for 6 weeks at indicated doses. For in vitro experiments, Rg3 was dissolved in dimethyl sulfoxide to prepare a high-concentration stock solution.

### Cell culture

AML12 cells were cultured in DMEM/F12 medium supplemented with 10% fetal bovine serum (FBS), insulin-transferrin-selenium, dexamethasone, and antibiotics at 37°C with 5% CO_2_. To isolate primary mouse hepatocytes, mice were anesthetized, and the liver was perfused *in situ* with EGTA-containing buffer followed by collagenase type IV digestion. The digested liver was minced, filtered through a cell strainer (70–100 μm), and centrifuged (50 × g, 5 min) to purify the hepatocytes. The cells were resuspended in Williams’ E medium supplemented with 10% FBS, insulin, dexamethasone, penicillin/streptomycin, and L-glutamine and maintained at 37°C with 5% CO_2_. The medium was replaced 4–6 h post-seeding to remove non-adherent cells.

### Biochemical assessment

Plasma levels of alanine aminotransferase (ALT), aspartate aminotransferase (AST), cholesterol (TC), triglycerides (TGs), lactate dehydrogenase (LDH), non-esterified fatty acids (NEFAs), high-density lipoprotein cholesterol (HDL-C), and low-density lipoprotein cholesterol (LDL-C) in mouse plasma were measured using an automated biochemical analyzer. The levels of IL-1β, IL-10, and TNFα in the plasma and livers of mice were determined according to the instructions provided on the ELISA kits (88-7013-22; 88-7324-22; 88-7064-22, Invitrogen, Waltham, MA, USA). Plasma OxLDL levels were measured using ELISA kit (LM-OxLDL-Mu, LMAI Bio, Shanghai, China) following the manufacturer’s instructions.

Hepatic and cellular levels of TG (E1013, Applygen), TC (BC1985, Applygen), MDA (BC0025, Solarbio), CAT (BC0205, Solarbio), SOD (BC5165, Solarbio), GSSG (BC1185, Solarbio), GSH (BC1175, Solarbio), and NADPH (BC1105, Solarbio) were measured using the corresponding assay kits according to the manufacturer’s instructions. The levels of Fe^2+^ were quantified using a commercial ELISA kit (Abcam，ab83366). The levels of OXPAPC in human and mice liver tissues were measured using a commercial ELISA kit (ML037449 and YJ589936, Shanghai, China).

### Hematoxylin and eosin (H&E) and Oil Red O (ORO) staining

Fresh liver tissue samples were fixed in 10% formalin at room temperature for 24 h. Sections of 4–5 μm thickness were cut and stained with H&E. After staining, the sections were dehydrated, cleared, and mounted for observation. For ORO staining, frozen liver sections were immersed in ORO staining solution for 10–15 min, and counterstained with hematoxylin. The sections were then dehydrated, cleared, and mounted. Lipid droplets were visualized using a microscope (Olympus BX41).

### Immunofluorescence (IF) and immunohistochemistry (IHC) Staining

For IF staining, paraffin-embedded tissue sections were deparaffinized and hydrated, followed by antigen-retrieved in citrate buffer. Sections were permeabilized with 0.1% Triton X-100 and blocked with 5% BSA, then incubated overnight at 4 °C with primary antibodies against GPX4, ACSL4, and F4/80. After washing, sections were incubated with Alexa Fluor-conjugated secondary antibodies for 1 h in the dark, and nuclei were counterstained with DAPI. For IHC staining, deparaffinized and hydrated sections underwent antigen retrieval and endogenous peroxidase blocking, followed by overnight incubation at 4 °C with a primary antibody against 4-HNE. Sections were subsequently incubated with a HRP-conjugated secondary antibody. Signals were visualized with DAB substrate and hematoxylin counterstaining. Images were captured microscopically (Olympus BX41), and positive staining was quantified via ImageJ software. Primary antibody information is listed in Table S1.

### Transmission electron microscopy

Fresh mouse liver tissues were cut into 1-mm^3^ cubes, or cell samples were pelleted by centrifugation and fixed in 2.5% glutaraldehyde for 4 h, followed by post-fixation with 1% osmium tetroxide for 1–2 h. Samples were dehydrated using a graded ethanol series and embedded in epoxy resin for polymerization. Ultra-thin sections (~70 nm) were prepared using an ultramicrotome, mounted onto copper grids, and stained with uranyl acetate and lead citrate. Sections were examined using a transmission electron microscope, and ultrastructural features were imaged and analyzed.

### Measurement of mtDNA copy number

Total DNA was isolated from mouse liver tissue using a DNA extraction kit (Cat# 4403319, Thermo Fisher Scientific) according to the manufacturer’s protocol. The mitochondrial DNA (mtDNA) content was quantified via quantitative polymerase chain reaction (qPCR) with primers specific to the mitochondrial genome.

### Western blotting

Cells or tissues were lysed using RIPA lysis buffer containing protease inhibitors to extract total protein, and the supernatant was collected for protein concentration determination. The extracted proteins were separated using SDS-PAGE, before transferring to a PVDF membrane. Membranes were incubated overnight at 4°C with primary antibodies specific to the indicated target proteins, followed by incubation with HRP-conjugated secondary antibodies at room temperature for 2 h. Protein bands were visualized using an imaging system. Primary antibody information is listed in Table S1.

### Micropyrokinetic technique

For the micropyrokinetic technique (MST) experiment, the equilibrium dissociation constant (Kd) values were measured using a Monolith NT.115 instrument (NanoTemper Technologies). Briefly, the SPOP protein was fluorescently labeled using the HIS-tag red channel (MO-L018), and Rg3 was diluted to the required concentrations (ranging from 200 µM to 0.0061 µM) and incubated with 0.1 µM of the fluorescently labeled SPOP protein for 15 min. The samples were loaded into NanoTemper glass capillaries (MO-K025) and subjected to microscale thermophoresis under a 40% light-emitting diode power. The Kd values were calculated from the repeated readings using the mass-action equation via the NanoTemper software. Each experiment was conducted at least three times.

### Co-Immunoprecipitation (Co-IP) and GST pull-down

For Co-IP, cells were lysed in RIPA buffer to extract total protein. Clarified lysates were incubated overnight at 4°C with the indicated primary antibodies or control IgG to allow immune complex formation. Protein A/G agarose beads were then added, and the mixture was incubated for an additional 2 h to capture the antibody-protein complexes. The beads were washed with wash buffer to remove nonspecifically bound proteins. Finally, the bound proteins were eluted and resolved by SDS-PAGE, and analyzed by western blotting. IgG controls were included in all experiments to assess nonspecific binding.

For GST-pull down assays, purified GST-SPOP fusion protein was immobilized on glutathione agarose beads, and unbound proteins were removed by washing. The beads were then incubated with lysates containing the indicated target proteins to allow protein-protein complexes to form. After extensive washing to remove nonspecific interactors, bound proteins were eluted using elution buffer, resolved by SDS-PAGE, and analyzed by western blotting. GST protein alone was used as a negative control.

### Mitochondrial oxygen consumption rate (OCR) in cells

Cells were seeded at 1 × 10^4^/well in a Seahorse XFe 96-well plate. After treatment, the medium was replaced with unbuffered Seahorse XF medium (pH 7.4) containing 10 mM glucose, 1 mM sodium pyruvate, and 2 mM GlutaMAX, followed by incubation at 37°C without CO_2_ for 1 h. Sequentially injected compounds (final concentrations: 1 μM oligomycin, 0.5 μM FCCP, 0.5 μM rotenone/antimycin A) were used to assess the basal respiration, maximal respiration, and non-mitochondrial oxygen consumption. OCR values were recorded using the XFe Analyzer and analyzed with Seahorse Wave software.

### Flow cytometric analysis

Cells were collected, washed with PBS, and stained with MitoSOX Red or BODIPY^™^ 581/591 C11 (37°C, 30 min, in the dark). Unstained cells were used as negative controls. After two washes with PBS, the cells were resuspended and analyzed via flow cytometry. The fluorescence intensity was quantified using FlowJo software, and the data were normalized to negative controls.

### Dual-Luciferase reporter assay

The NUPR1 promoter fragment was subcloned into the pGL3-Basic vector to generate the firefly luciferase reporter construct. Human embryonic kidney 293T cells were seeded in 24-well plates and co-transfected with the reporter plasmid, an ATF3 overexpression plasmid or an empty vector (negative control), and the pRL-TK Renilla luciferase plasmid (internal reference) using Lipofectamine 3000. After 48 h of transfection, luciferase activities were measured with the Dual-Luciferase Reporter Assay System. Relative luciferase activity was calculated as the ratio of firefly luciferase activity to Renilla luciferase activity.

### RNA sequencing

RNA sequencing was performed by OE Biotech, Inc., Shanghai, China. Briefly, total RNA was extracted from mouse liver tissues, and RNA quantity and quality were assessed using a Qubit 4.0 for concentration measurement and an Agilent Bioanalyzer for fragment size distribution and integrity evaluation. Sequencing libraries were normalized and sequenced on the Illumina NovaSeq 6000 platform in paired-end mode (PE150), generating 10–20 million reads per sample to ensure coverage of low-abundance transcripts. The raw data quality was evaluated using FastQC, and low-quality reads/adapters were filtered using Trimmomatic. Clean reads were aligned to the reference genome via HISAT2 or STAR. Gene/transcript expression levels (FPKM or TPM) were quantified using StringTie or Salmon. Differentially expressed genes were identified using DESeq2 or edgeR, with significance determined by an adjusted p-value (or False Discovery Rate, FDR) < 0.05. Functional enrichment analysis (GO and KEGG pathways) was performed on the significant gene set using clusterProfiler or DAVID, and pathways with a q-value < 0.05 were considered significantly enriched

### Proteomics analysis

Proteomics analysis was performed using a data-independent acquisition (DIA) workflow. Briefly, proteins extracted from liver tissues were digested with trypsin, desalted, and separated by reversed-phase ultra-high-performance liquid chromatography (UHPLC, Thermo Vanquish Neo system) using a 50 cm Low-Load µPAC^™^ Neo HPLC Column. Mobile phases A (0.1% formic acid in water) and B (0.1% formic acid in 80% acetonitrile) were delivered at 2.2 μL/min with an 8-min linear gradient. Peptide analysis was performed on an Orbitrap Astral mass spectrometer operated in DIA mode: full MS scans (380–980 m/z, resolution: 240,000, AGC: 500%, injection time: 5 ms) and DIA MS/MS scans (150–2000 m/z, isolation window: 2 m/z, AGC: 500%, injection time: 3 ms) with 25% collision energy and a 0.6 s cycle time. Full MS and DIA spectra were recorded in profile and centroid modes, respectively. Raw data were processed using MaxQuant or Spectronaut (UniProt database) with a precursor mass tolerance ±10 ppm and trypsin/P specificity (≤ 2 missed cleavages). Differentially expressed proteins were identified based on a fold change ≥ 1.5 and an adjusted *p*-value < 0.05. These proteins were then subjected to functional enrichment analysis using DAVID and STRING, where enriched terms were considered significant if the Benjamini-Hochberg adjusted *p*-value < 0.05.

### Immunoprecipitation-mass spectrometry (IP-MS) analysis

For IP-MS analysis, proteins were extracted from AML12 cells pretreated with chloroquine and MG132 for 6 h prior to OxPAPC exposure. Cell lysates were incubated with an anti-SPOP antibody conjugated to protein A/G agarose beads to form antigen-antibody complexes. As a negative control, parallel immunoprecipitations were performed using isotype-matched IgG. The immune complexes were isolated using the beads and washed extensively to remove nonspecifically bound proteins. Bound proteins were eluted and subjected to LC-MS/MS analysis for protein identification and quantification. Data were processed using MaxQuant or a similar platform to determine the label-free quantification (LFQ) intensity for each protein. To identify high-confidence interacting proteins, a two-sample t-test was performed comparing the LFQ intensities between SPOP-IP and IgG-IP replicates. The resulting p-values were adjusted for multiple testing using the Benjamini-Hochberg method. Proteins were considered bona fide interactors if they met the criteria of an FDR < 0.05 and a fold-enrichment > 2.

### LC-MS/MS-based oxylipidomics analysis

Tissue samples were homogenized in 750 µL of chloroform: methanol: MilliQ H_2_O (3:6:1) (v/v/v) and incubated at 4°C with gentle agitation (1500 rpm) for 1 h. Phase separation was induced by adding 350 µL of deionized water and 250 µL of chloroform, followed by centrifugation. The lower organic phase containing lipids was collected into a clean tube. To maximize lipid recovery, the extraction was repeated once by adding 450 µL of chloroform to the remaining aqueous phase. Organic extracts were pooled and dried under vacuum using a SpeedVac in OH mode. Lipidomic analyses were conducted at LipidALL Technologies using an ExionLC-AD—Sciex QTRAP 6500 PLUS. Separation of oxidized phospholipid classes was achieved by normal-phase (NP) HPLC using a TUP-HB silica column (i.d. 150 mm × 2.1 mm, 3 µm). Multiple reaction monitoring (MRM) transitions were set up for comparative analysis of various oxidized phospholipids. Individual lipid species were quantified by referencing spiked internal standards. DMPC and DMPE were obtained from Avanti Polar Lipids. The resulting p-values were then adjusted for multiple comparisons using the Benjamini-Hochberg procedure. Lipids with an FDR < 0.05 were considered significantly altered.

### Chromatin Immunoprecipitation sequencing (ChIP-seq) analysis

Cells were fixed with 1% formaldehyde to crosslink DNA-protein complexes, followed by glycine quenching. Cells were lysed, and chromatin was fragmented by sonication to an average size of 200–500 bp. Fragmented chromatin was immunoprecipitated overnight at 4°C with an ATF3-specific antibody and Protein A/G magnetic beads (Thermo Fisher Scientific). An isotype-matched IgG immunoprecipitation was performed in parallel and served as the background control. After washing, the complexes were eluted and reverse crosslinked. DNA was purified, and sequencing libraries were prepared and sequenced. Raw data were subjected to quality control, adapter trimming, and alignment to the reference genome (mm10, Bowtie2). Peak calling was performed using MACS2 by comparing the ATF3 ChIP-seq data against the IgG control data, and significant peaks were identified using a FDR threshold of < 0.05. Motif analysis (MEME Suite) and visualization (Integrative Genomics Viewer, IGV) were subsequently performed on the significant peak set.

### Statistical analysis

All quantitative data are presented as mean ± SEM, where *n* indicates the number of biological replicates. Statistical analyses were performed using GraphPad Prism 9 (GraphPad, La Jolla, CA, USA). The data were analyzed using Student's t-test for two-group comparisons, and one-way ANOVA followed by Tukey's multiple comparisons tests for comparisons involving three or more independent groups where appropriate. For repeated measures data, a linear mixed-effects model (LMM) was applied, post-hoc pairwise comparisons were performed using Tukey's multiple comparisons test after LMM analysis. A *p*-value < 0.05 was considered statistically significant for all tests.

**Figure S1 High-dose Rg3 ameliorates hyperlipidemia and inflammatory injury in nonalcoholic steatohepatitis (NASH) mice.** (A-C) TG (A), TC (B), and NEFA (C) contents in the plasma of mice, n = 8. (D and E) LDL-C (D) and HDL-C (E) contents in the plasma of mice, n = 8. (F-I) Quantification of HE (F), ORO (G), PSR (H), and CIV (I) positive cells, n = 6. (J-O) IL-6 (J, K), TNFα (L, M), and IL-1β (N, O) contents in the plasma and liver of mice, n = 8. Data are shown as the mean ± SEM; **p* < 0.05, ***p* < 0.01.

**Figure S2 Rg3 alleviates ferroptosis and upregulates hepatic *Spop* expression.** (A) Heatmap indicating changes to different genes in mice liver, n = 5. (B) Heatmap indicating changes to different proteins in mice liver. (C) KEGG pathway enrichment analysis of mouse hepatic proteomic data. (D-F) MDA (D), CAT (E), and SOD (F) content in the livers of mice, n = 8. (G-I) GSH (G), GSSG (H), and GSH/GSSG (I) content in the livers of mice, n = 8. (J) Quantification of DAB Perls’ Blue/iron stanning positive cells, n = 6. (K) OxPAPC content in the livers of patients, n = 3. (L) PCA of oxylipidomics in the mice livers, n = 5. (M) IHC detection and quantitative analysis of positive cells for 4-hydroxynonenal (4-HNE), n = 6. (N) Representative images acquired via transmission electron microscopy. MT: Mitochondria, LD: Lipid drop. (O) Protein-protein interaction network (HFD vs. HFD/Rg3). (P) Gene expression of *Spop* in mice, n = 8. Data are shown as the mean ± SEM; **p* < 0.05, ***p* < 0.01.

**Figure S3 SPOP and Rg3 molecular docking.** (A) Gene expression of *Spop* in AML12 cells, n = 3. (B) Protein expression of SPOP in AML12 cells, n = 3. (C) Effect of different cell death inhibitors on the cell viability of AML12 cells exposed to OxPAPC for 24 h. All inhibitors were added for pretreatment for 2 h before OxPAPC treatment. Ferroptosis inhibitors: ferrostatin-1 (Fer-1, 1 μM), deferoxamine (DFO, 100 μM); necroptosis inhibitor: necrostatin-1 (Nec-1, 1 μM); and apoptosis inhibitor: Z-VAD-FMK (ZVAD, 1 μM). (D) Root means square deviation (RMSD) of Rg3 and SPOP. (E) Solvent accessible surface area (SASA) of the protein and small molecule. (F) the number of hydrogen bonds between the protein and small molecule. (G) Cell viability assayed by CCK-8, n = 3. Data are shown as the mean ± SEM; **p* < 0.05, ***p* < 0.01.

**Figure S4 *Spop* interference impairs the ferroptosis-resistant effect of Rg3.** (A-F) GSH (A), GSH/ GSSG (B), SOD (C), CAT (D), MDA (E), and NADPH (F) content in AML12 cells, n = 3. (G) Protein expression of GPX4 in AML12 cells, n = 3. (H) JC-1 of AML12 cells, n = 3. (I) Representative images acquired via transmission electron microscopy, n = 3. (J) DNA copy number of AML12 cells, n = 3. Data are shown as the mean ± SEM; **p* < 0.05, ***p* < 0.01.

**Figure S5 CO-IP between SPOP and ATF3.** (A) Protein expression of GPX4 in AML12 cells, n = 3. (B and C) Co-IP of SPOP (B) and ATF3 (C) in AML12 cells, n = 3. Data are shown as the mean ± SEM; **p* < 0.05, ***p* < 0.01.

**Figure S6** **Overexpression of *Spop* ameliorates *Atf3*-induced hepatocyte ferroptosis.** (A) Validation of siRNA and plasmid transfection efficiency in AML12 cells, n = 3. (B) Cells overexpressing *Spop* were transfected with HA-tagged plasmids, followed by detection of K63-linked ubiquitination of ATF3, n = 3. (C-E) GSH (C), MDA (D) and SOD (E) content in AML12 cells, n = 3. (F) Protein expression of GPX4 in AML12 cells, n = 3. Data are shown as the mean ± SEM; **p* < 0.05, ***p* < 0.01.

**Figure S7** **The distribution of ChIP-seq peaks and the top five motifs.** (A) The distribution of ChIP-seq peaks, Reptea1, Repeat2, and Repeat3, corresponds to three technical replicates. (B) The top five motifs.

**Figure S8** **Enrichment analysis of ATF3 target genes.** (A) ChIP-seq TSS plot. (B) KEGG enrichment analysis of ATF3 target genes. (C) GO enrichment analysis of ATF3 target genes. (D) ChIP-seq tracks of ATF3 binding to *Slc7a11*, *Sqor*, and *Fxn*.

**Figure S9 The effect of ATF3 interference on the expression of candidate genes.** (A) Gene expression of *Nupr1* in AML12 cells, n = 3. (B) Protein expression of NUPR1 in AML12 cells, n = 3. (C-E) Gene expression of *Slc7a11* (C), *Fxn* (D), and *Sqor* (E) in AML12 cells, n = 3. (F-H) Protein expression of SLC7A11, and FXN in AML12 cells, n = 3. Data are shown as the mean ± SEM; **p* < 0.05, ***p* < 0.01.

**Figure S10** **SPOP, NUPR1 and ATF3 expression in the liver of patients with cirrhosis.** (A and B) PSR (A) and DAB Perls’ Blue/iron positive cell staining (B) on liver sections of patients with cirrhosis, n = 6. (C and D) Protein expression (C) and immunofluorescence (IF) (D) staining of SPOP, ATF3, and NUPR1 in patients with cirrhosis, n = 3. Data are shown as mean ± SEM; **p* < 0.05, ***p* < 0.01.

**Figure S11** ***Nupr1* rescues the impaired anti-ferroptotic function of Rg3 caused by *Spop* knockdown in hepatocyte.** (A and B) Lipid ROS detected via flow cytometry after staining AML12 cells with BODIPY, n = 3. (C and D) Lipid ROS detected via flow cytometry after staining BRL3A cells with BODIPY, n = 3. (E-G) GSH (E), MDA (F), and SOD (G) content in AML12 cells, n = 3. (H-J) GSH (H), MDA (I), and SOD (J) content in BRL3A cells, n = 3. (K) Representative images of transmission electron microscopy in AML12 cells, n = 3. (L) Representative images of transmission electron microscopy in BRL3A cells, n = 3. (M) Mitochondria of AML12 cells were subjected to bioenergetics analysis using the Seahorse Bioscience XF analyzer, n = 3. (N) Mitochondria of BRL3A cells were subjected to bioenergetics analysis using the Seahorse Bioscience XF analyser, n = 3. (O) Protein expression of ACSL4 and GPX4 in AML12 cells, n = 3. (P) Protein expression of ACSL4 and GPX4 in BRL3A cells, n = 3. Data are shown as the mean ± SEM; **p* < 0.05, ***p* < 0.01.

**Figure S12 Morphological detection in mouse CCL4 model.** (A and B) Protein expression of SPOP (A) and NUPR1 (B) in mice, n = 6. (C) SOD, and MDA content in primary hepatocytes of CCL4 model mice, n = 6. (D) ALT, and AST levels in plasma of mice (CCL4 model), n = 6. (E) Representative images of enhanced DAB Perls’ Blue/iron staining and quantification of positive cells (CCL4 model), n = 6. (F) OxPAPC content in the plasma of mice, n = 6. (G) Basal respiration, n = 3. (H) ATP-coupled respiration, n = 3. (I) GPX4 fluorescence intensity in liver of CCL4 model mice, n = 6. (J and K) IF staining of ACSL4 (J) and F4/80 (K) in the liver of mice (CCL4 model), n = 6. Data are shown as the mean ± SEM; **p* < 0.05, ***p* < 0.01.

**Figure S13** **Overexpression of *Nupr1* rescues the attenuation of Rg3-mediated anti-ferroptotic effects caused by *Spop* knockdown in mouse OxPAPC model.** (A) Schematic depicting the Rg3 treatment strategy in the OxPAPC-induced hepatocyte ferroptosis model mice. (B-D) GSH (B), SOD (C), and MDA (D) content in primary hepatocytes of HFD model mice, n = 6. (E) Iron content in the livers of mice, n = 6. (F) OxPAPC content in the livers of mice, n = 6. (G) Lipid ROS detected via flow cytometry after staining primary hepatocytes of OxPAPC model mice with BODIPY, n = 3. (H) Mitochondria in primary hepatocytes of OxPAPC model mice were subjected to bioenergetics analysis using the Seahorse Bioscience XF analyzer, n = 3. (I) IF staining of GPX4 in liver of OxPAPC model mice, n = 6. Data are shown as the mean ± SEM; **p* < 0.05, ***p* < 0.01.

**Figure S14 Morphological detection in mouse OxPAPC model.** (A and B) AST (A), and ALT (B) levels in plasma of mice (OxPAPC model), n = 6. (C) OxPAPC content in the plasma of mice, n = 6. (D) Representative images of enhanced DAB Perls’ Blue/iron staining and quantification of positive cells (OxPAPC model), n = 6. (E) IF staining of GPX4 in the liver of mice (OxPAPC model), n = 6. (F) IF staining of GPX4 in the liver of mice (OxPAPC model), n = 6. Data are shown as the mean ± SEM; **p* < 0.05, ***p* < 0.01.
